# Myeloid and T-Cell Microenvironment Immune Features Identify Two Prognostic Sub-Groups in High-Grade Gastroenteropancreatic Neuroendocrine Neoplasms

**DOI:** 10.3390/jcm10081741

**Published:** 2021-04-17

**Authors:** Giovanni Centonze, Vincenzo Lagano, Giovanna Sabella, Alessandro Mangogna, Giovanna Garzone, Martina Filugelli, Beatrice Belmonte, Laura Cattaneo, Valentina Crisafulli, Alessio Pellegrinelli, Michele Simbolo, Aldo Scarpa, Paola Spaggiari, Tatiana Brambilla, Sara Pusceddu, Natalie Prinzi, Andrea Anichini, Claudio Tripodo, Massimo Milione

**Affiliations:** 1 1st Pathology Division, Department of Pathology and Laboratory Medicine, Fondazione IRCCS Istituto Nazionale dei Tumori, 20133 Milan, Italy; giovanni.centonze@istitutotumori.mi.it (G.C.); vincenzo.lagano@istitutotumori.mi.it (V.L.); giovanna.sabella@unimi.it (G.S.); giovanna.garzone@istitutotumori.mi.it (G.G.); martina.filugelli@istitutotumori.mi.it (M.F.); laura.cattaneo@istitutotumori.mi.it (L.C.); valentina.crisafulli@istitutotumori.mi.it (V.C.); 2 Department of Research, Fondazione IRCCS Istituto Nazionale dei Tumori, 20133 Milan, Italy; andrea.anichini@istitutotumori.mi.it; 3 Institute for Maternal and Child Health, IRCCS Burlo Garofalo, 34137 Trieste, Italy; alessandro.mangogna@burlo.trieste.it; 4 Tumor Immunology Unit, University of Palermo, 90133 Palermo, Italy; beatrice.belmonte@unipa.it (B.B.); tripodo@unipa.it (C.T.); 5 Department of Pathology, ASST Franciacorta, Mellino Mellini Hospital, 25032 Brescia, Italy; alessio.pellegrinelli@asst-franciacorta.it; 6 Department of Diagnostics and Public Health, Section of Pathology, University of Verona, 37134 Verona, Italy; simbolo.michele@hotmail.it (M.S.); aldo.scarpa@univr.it (A.S.); 7 ARC-NET Centre for Applied Research on Cancer, University of Verona, 37134 Verona, Italy; 8 Department of Pathology, IRCCS Humanitas Research Hospital, 20089 Rozzano, Italy; paola.spaggiari@humanitas.it (P.S.); tatiana.brambilla@humanitas.it (T.B.); 9 Medical Oncology Department, Fondazione IRCCS Istituto Nazionale dei Tumori, 20133 Milan, Italy; sara.pusceddu@istitutotumori.mi.it (S.P.); natalie.prinzi@istitutotumori.mi.it (N.P.)

**Keywords:** gastroenteropancreatic neuroendocrine neoplasms, tumor microenvironment, myeloid markers

## Abstract

High-grade Gastroenteropancreatic Neuroendocrine neoplasms (H-NENs) comprehend well-differentiated tumors (NET G3) and poorly differentiated carcinomas (NEC) with proliferative activity indexes as mitotic count (MC) >20 mitoses/10 HPF and Ki-67 >20%. At present, no specific therapy for H-NENs exists and the several evidences of microenvironment involvement in their pathogenesis pave the way for tailored therapies. Forty-five consecutive cases, with available information about T-cell, immune, and non-immune markers, from surgical pathology and clinical databases of 2 Italian institutions were immunostained for Arginase, CD33, CD163 and CD66 myeloid markers. The association between features was assessed by Spearman’s correlation coefficient. A unsupervised K-means algorithm was used to identify clusters of patients according to inputs of microenvironment features and the relationship between clusters and clinicopathological features, including cancer-specific survival (CSS), was analyzed. The H-NEN population was composed of 6 (13.3%) NET G3 and 39 (86.7%) NEC. Overall, significant positive associations were found between myeloid (CD33, CD163 and Arginase) and T/immune markers (CD3, CD4, CD8, PD-1 and HLA-I). Myeloid and T-cell markers CD3 and CD8 identified two clusters of patients from unsupervised K-means analysis. Cases grouped in cluster 1 with more myeloid infiltrates, T cell, HLA and expression of inhibitory receptors and ligands in the stroma (PD-1, PD-L1) had significantly better CSS than patients in cluster 2. Multivariable analysis showed that Ki-67 (>55 vs. <55, HR 8.60, CI 95% 2.61–28.33, *p* < 0.0001) and cluster (1 vs. 2, HR 0.43, CI 95% 0.20–0.93, *p* = 0.03) were significantly associated with survival. High grade gastroenteropancreatic neuroendocrine neoplasms can be further classified into two prognostic sub-populations of tumors driven by different tumor microenvironments and immune features able to generate the framework for evaluating new therapeutic strategies.

## 1. Introduction

According to World Health Organization (WHO) 2019, High-Grade Gastroenteropancreatic neuroendocrine neoplasms (H-NENs) comprehend tumors with a Ki-67 index >20% distinguished in NET-G3, when Well-Differentiated (WD), or NEC-G3, when Poorly-Differentiated (PD) [1]. Among these, NEC-G3s are characterized by the worst prognosis [2]. Accurate diagnosis of these diseases is critical for correct prognosis and clinical management. High-grade G3 NET and NEC need a complex therapeutic approach due to a lack of clinical data and low response to treatments; to date, there are no consensus guidelines for the management of these rare and aggressive neoplasms [3,4,5]. Moreover, NECs are often diagnosed at a later stage and treated with platinum-etoposide chemotherapy with fractional efficacy [6,7,8]. To the best of our knowledge, no reliable prognostic marker, able to predict clinical outcome, has been definitively validated to date. Therefore, clinical management of these neoplasms is still controversial.

The tumor microenvironment (TME) is a dynamic compartment that develops during cancer evolution, constituted by tumor, immune, stromal and inflammatory cells, cancer-associated fibroblasts (CAFs), vasculature and extracellular matrix (ECM) [9,10]. TME has been on the spotlight of cancer research in recent years for its pivotal role in tumorigenesis and cancer progression as well as in regulating the efficacy of the therapeutic response in neuroendocrine neoplasms [11,12,13,14]. In our recent work, we demonstrated that microenvironment-related immune and inflammatory markers can improve prognostic prediction in GEP-NENs, when combined with the established Ki-67 and morphology parameters [15]. The aim of this study is to further evaluate the tumor microenvironment of high-grade neuroendocrine neoplasms, by expanding the immune profiling to myeloid markers Arginase, CD33, CD163 and CD66.

## 2. Materials and Methods

### 2.1. Cases

Forty-five consecutive patients among the prospectively maintained clinical databases of 2 Italian institutions (Fondazione IRCCS Istituto Nazionale dei Tumori and Humanitas Research Hospital Rozzano) were retrospectively selected. Formalin-fixed and paraffin-embedded (FFPE) specimens collected by these patients were reviewed according to the rules described in the following paragraph. In order to correlate and investigate myeloid markers with the content of tumor microenvironment, we used data regarding T-cell (CD3, CD4, CD8), immune (PD-1, PD-L1, HLA-I, HLA-DR) and non-immune markers (COX-2, pS6, β-catenin, NGFR, α-SMA, CD31) of our previous work [15].

The study was performed according to the clinical standards of the 1975 and 1983 Declaration of Helsinki and was approved by the Ethics Committee of Fondazione IRCCS INT (No. INT 21/16, 28 January 2016).

### 2.2. Immunohistochemistry

Arginase, CD33, CD163 and CD66 were investigated by IHC methods. Briefly, sections 2.5/3 micron-thick were deparaffinized and rehydrated. The antigen unmasking technique was carried out using Novocastra Epitope Retrieval Solutions, pH 6 EDTA-based (Leica Biosystems, Wetzlar, Germany) in a thermostatic bath at 98° for 30 min. Sections were then brought to room temperature and washed in PBS. After neutralization of the endogenous peroxidase with 3% *v*/*v*, H_2_O_2_ and Fc blocking by a specific protein block (Novocastra, Leica Biosystems), the samples were incubated with primary antibodies listed in Supplementary Table S1. Staining was revealed using a Novolink Polymer Detection System (Novocastra) and AEC (3-Amino-9-ethylcarbazole) as a substrate-chromogen, following the manufacturer’s instructions. Slides were counterstained with Harris Hematoxylin (Novocastra, Leica Biosystems). Slides were analyzed under the Axio Scope A1 optical microscope (Zeiss Oberkochen, Germany) and microphotographs were collected through the Axiocam 503 color digital camera (Zeiss) using the Zen2 software (Supplementary Figure S1).

IHC was assessed first by two expert pathologists, one in a neuroendocrine (M.M.) and one in a microenvironment (C.T.) field. As previously described, to minimize the variability of the evaluation, IHC was evaluated with a semi-quantitative approach, by adopting a scoring system that takes into account both the intensity and extent of the staining marker (% of positive cells) [15,16]. Briefly, the intensity (I) was ranked as low (1+; fainter than internal controls), normal (2+; as faint as controls), or strong (3+; more intense than controls). The expression (E) was defined as follows: up to 25% cells, 1+; 26–50%, 2+; 51–75%, 3+; 76–100%, 4+. I and E were combined into a single score (S), calculated as I × E, ranging from 0 to 12. Specimens were directly included only in the case of full consensus by both aforementioned expert pathologists; furthermore, in cases in which consensus was not reached, a majority decision was adopted during panel consensus meetings composed of five additional pathologists (A.P.; B.B.; L.C.; P.S.; T.B.). The evaluation of myeloid markers was carried out specifically in the stromal cells and median cut-offs were used for survival analysis.

### 2.3. Statistical Analysis

The association between all the investigated features was assessed by Spearman’s correlation coefficient. Unsupervised K-means algorithm [17] was used to identify clusters of patients according to inputs of microenvironment stromal features. Briefly, the algorithm works first by randomly assigning centroids and then by calculating the distance of each data point to its nearest centroid using the Euclidean distance. Subsequently, it finds the new value of centroids by calculating the mean distance of all points belonging to the centroid. Each data point is grouped into a cluster with the minimum distance to the centroid of the cluster and maximum inter-cluster distance by an iterative process. The data were also subjected to principal component analysis (PCA) and a biplot was produced to investigate the relationships between two principal components and microenvironment features.

The association between morphological and immunophenotypical features and clusters was assessed using Fisher’s exact test for categorical variables and Wilcoxon’s rank sum test for continuous variables. Cancer-specific survival (CSS) was assessed from the time of the diagnosis to the time of disease-related death or last follow-up, whichever occurred first. CSS curves were drawn using the Kaplan–Meier method. The log-rank test was used to assess the survival difference between the patient groups. Cox proportional regression analysis was used to assess the association between morphological and immunophenotypical features and survival. Hazard ratios (HRs) are presented with respective 95% confidence interval (CI). Data analysis were performed using the R environment for statistical computing and graphics (R Foundation, Vienna, Austria-Version 3.6.2). All tests were two-sided and *p*-values <0.05 were considered statistically significant.

## 3. Results

### 3.1. Patient Characteristics and Myeloid Profile

Table 1 summarizes the clinico-pathological features of the 45 patients included in the study. Overall, the cohort comprised 28 (62.2%) males and 17 (37.8%) females with a median age of 61 years with a range 33–78. The most represented tumor site was the pancreas (*n* = 15, 33.3%), followed by rectum (*n* = 10, 22.2%), colon (*n* = 8, 17.8%), ileum (*n*= 7, 15.6%) and stomach (*n* = 5, 11.1%). A pathological review, using both morphology and Ki-67 55% cut-off, classified them in 6 (13.3%) NET G3, 8 (17.8%) NEC Ki-67 <55 and 31 (68.9%) NEC Ki-67 >55%.

**Figure 1:**
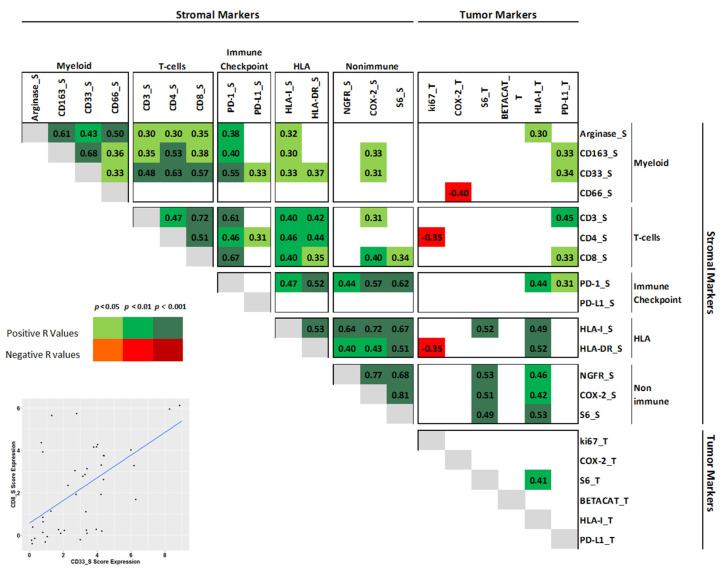
Binary associations of all investigated markers by Spearman correlation analysis. Only significant r values (either positive or negative) are shown. Expression of each marker was evaluated in the tumor (superscript T) or in the stroma (superscript S). On the bottom left: a representative scatter plot of CD8^S^ and CD33^S^ marker expression.

Myeloid markers CD33^S^, CD163^S^ and Arginase^S^ expressions showed a significant positive association with T-cell markers (CD3^S^, CD4^S^, CD8^S^), PD-1^S^ and HLA-I^S^ (Figure 1). In particular, CD33^S^ showed a strong positive association (*p* < 0.001) with T-cell CD3^S^ (r = 0.48), CD4^S^ (r = 0.63) and CD8^S^ (r = 0.57) and PD-1^S^ (r = 0.55) and mild (p-value from 0.05 to 0.02) with COX-2^S^ (r = 0.31), HLA-I^S^ (r = 0.33), HLA-DR^S^ (r = 0.37) and PD-L1^S^ (r = 0.33). Moreover, CD163^S^ showed a strong positive association (*p* < 0.001) with CD4^S^ (r = 0.53), while moderate associations (p-value from 0.05 to 0.002) were observed between CD163^S^ and Arginase^S^ with CD3^S^ (r = 0.35 and 0.30), CD8^S^ (r = 0.38 and 0.35), PD-1^S^ (r = 0.40 and 0.38) and HLA-I^S^ (r = 0.30 and 0.32). Interestingly, we observed also a mild positive association between CD33^S^ and CD163^S^ with tumor PD-L1^T^ (r = 0.34 and 0.33) and a negative association between CD66^S^ with COX-2^T^ (r = −0.40) expressed in neoplastic cells.

### 3.2. Clustering

**Figure 2:**
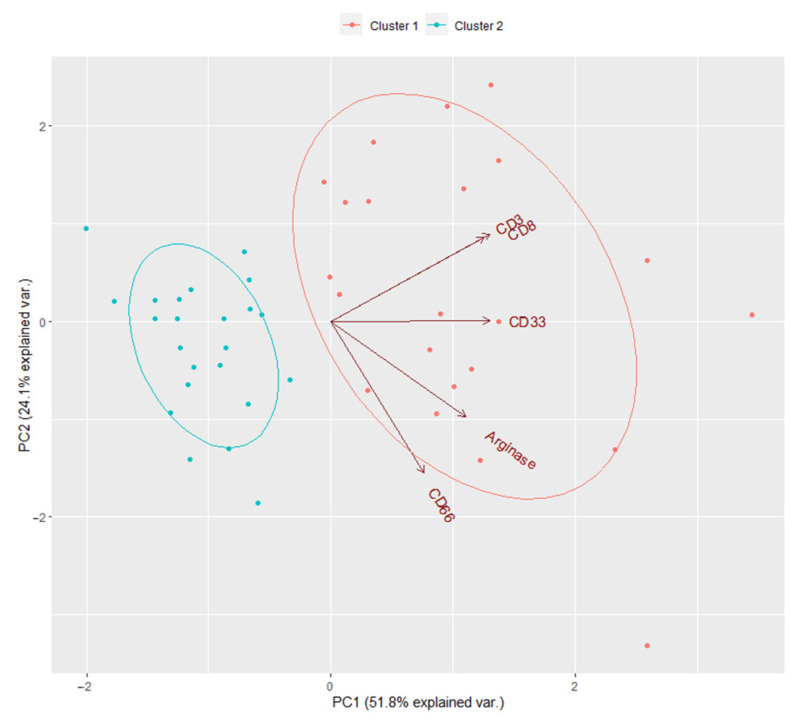
The biplot of unsupervised K-means analysis for myeloid Arginase^S^, CD33^S^ and CD66^S^ and T-cell CD3^S^ and CD8^S^.

Myeloid markers (Arginase^S^, CD33^S^ and CD66^S^) and T-cell markers CD3^S^ and CD8^S^ identified two clusters of patients by unsupervised K-means analysis with different features in the tumor microenvironment (Figure 2). Specifically, cluster 1 showed both higher myeloid and T-cell markers compared to cluster 2. PCA showed that the first two components explain about 75.9% of overall variability. First principal component (PC1) explained 51.8% and was significantly correlated mainly with CD8^S^, CD33^S^ and CD3^S^ while the second (PC2) explained 24.1% and correlated mainly with Arginase^S^ and CD66^S^.

**Figure 3:**
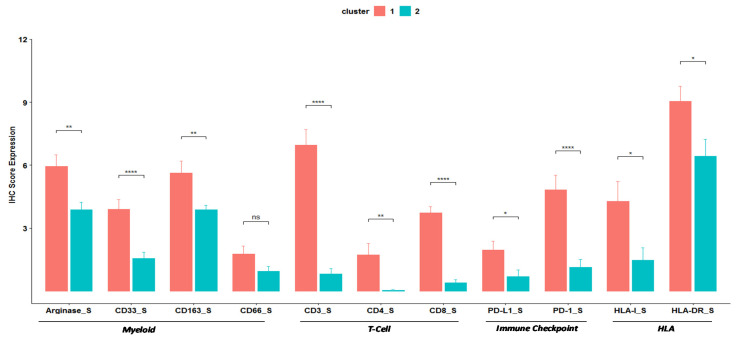
Barplots of immunohistochemistry Score Expression of Myeloid, T-cells, Immune checkpoint and HLA markers. * indicates the level of significance.

In-depth analysis of the two clusters revealed further specific differences in the expression of several markers (Figure 3, Supplementary Table S2). Cases grouped in cluster 1 included 5 (22. 7%) NET G3, 5 (22.7%) NEC Ki-67 <55 and 12 (54.6%) NEC Ki-67>55 while cluster 2 included 1 NET G3 (4.4%), 3 (13.0%) NEC Ki-67 <55 and 19 NEC Ki-67 >55 (82.6%). Overall, cases in cluster 1 showed more myeloid infiltrates, lymphoid T cell, HLA antigens and inhibitory receptors and ligands in the stroma compared to cluster 2 (Figure 3, Supplementary Table S2). Specifically, a significant increase in score estimated for myeloid Arginase^S^ (mean 5.95 (3–12) vs. 3.87 (0–9), *p* = 0.002), CD33^S^ (mean 3.90 (1–9) vs. 1.57 (0–4), *p* < 0.0001) and CD163^S^ (mean 5.64 (3–12) vs. 3.87 (2–6), *p* = 0.008) were observed in cluster 1. Moreover, CD66^S^ showed an increase (mean 1.77 (0–6) vs. 0.96 (0–3) *p* = 0.11), but without statistical differences.

A similar trend was observed in the estimated score for lymphoid T-cell, immune checkpoint and HLA markers: CD3^S^ (6.95 (1–12) vs. 0.83 (0–4), *p* < 0.0001), CD4^S^ (1.73 (0–9) vs. 0 (0–1), *p* = 0.001), CD8^S^ (3.73 (1–6) vs. 0.39 (0–2), *p* < 0.0001), PD-L1^S^ (1.95 (0–6) vs. 0.7 (0–6), *p* = 0.01), PD-1^S^ (4.82 (0–9) vs. 1.13 (0–6), *p* = 0.0002), HLA-I^S^ (4.27 (0–12) vs. 1.48 (0–9), *p* = 0.01) and HLA-DR^S^ (9.05 (2–12) vs. 6.43 (0–12), *p* = 0.02) were enriched in cluster 1 compared to cluster 2. On the other hand, no significant changes were observed between clusters for non-immune markers in the stroma and tumor markers (Supplementary Table S2). Thus, for all significant differences, expression of the informative markers was higher in cluster 1 than in cluster 2.

### 3.3. Survival Analysis

**Figure 4:**
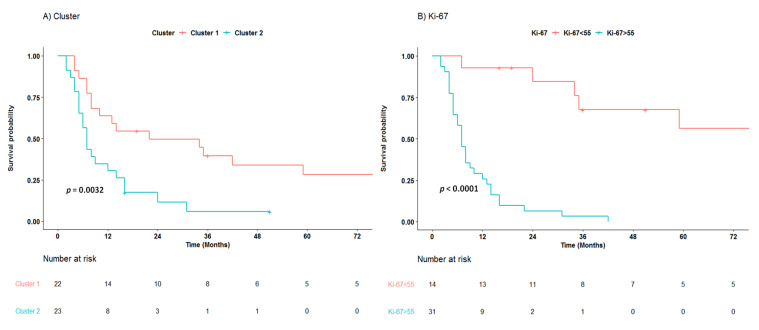
Cancer-specific survival of patients with high-grade neuroendocrine neoplasms according cluster (**A**) and Ki-67 55% cut off (**B**).

In the overall cohort Median CSS (mCSS) was 10 months (95% CI 7–24) (Supplementary Figure S2). In particular, mCSS was 22 (95% CI 10–92) for cluster 1 and 7 (95% CI 5–16) for cluster 2. Kaplan–Meier analysis patients grouped in cluster 1 had significantly better survival than patients in cluster 2 (log-rank *p* = 0.003; Figure 4A). This suggests that the more pronounced immune infiltration observed in lesions from cluster 1 reflect an ongoing immune response that has prognostic significance. Furthermore, cases with Ki-67 <55% (both NET G3 and NEC), had a better survival compared to Ki-67 ≥55% (*p* = <0.0001; Figure 4B).

Supplementary Table S3 shows results of Cox proportional regression analysis. After adjustment for tumor site, Ki-67 was strongly associated with survival (>55 vs. <55, HR 10.7, 95% CI 3.34–34.26, *p* < 0.001). However, interestingly, even distinct immune markers showed a prognostic significance. This included Arginase^S^ (4–12 vs. 0–3, HR 0.40, 95% CI 0.18–0.91, *p* = 0.03), CD3^S^ (3–12 vs. 1–2, HR 0.31, 95% CI 0.15–0.64, *p* = 0.001), CD8^S^ (2–12 vs. 0–1, HR 0.45, 95% CI 0.22–0.92, *p* = 0.03) and PD-1^S^ (2–12 vs. 0–1, HR 0.33, 95% CI 0.15–0.71, *p* = 0.005). Patients in cluster 1 decreased significantly the Hazard Ratio (HR) by factor 0.30 (CI 95% 0.14–0.63, *p* = 0.001) compared to patients in cluster 2. Moreover, multivariable analysis showed that Ki-67 (>55 vs. <55, HR 8.60, CI 95% 2.61–28.33, *p* < 0.0001) and cluster (1 vs. 2, HR 0.43, CI 95% 0.20–0.93, *p* = 0.03) were significantly associated with survival.

## 4. Discussion

Patients affected by High-grade Neuroendocrine Neoplasms (H-NENs) represent an heterogeneous and poorly understood population. We demonstrated that TME markers were able to further identify among H-NENs two cohorts showing different clinical outcomes according to HLA-I^T^, lymphoid markers CD3^S^ and CD8^S^ and PD-L1^S^ immunohistochemical expression_._ [15]. The current study further enriched H-NENs TME characterization through the assessment of myeloid markers Arginase, CD33, CD163 and CD66 expression. The present results showed that both myeloid and T cell markers share their expression in H-NENs. Furthermore, the aforementioned myeloid markers were capable to further describe H-NENs clinical outcome defining two well characterized H-NENs prognostic classes paving the way to propose the TME role as an independent clinical outcome predictor in H-NENs on the basis of the “hot/cold tumor” idea [18,19]. In detail, H-NENS rich in immune infiltrate should cover different immune subsets belonging to the innate (such as myeloid cells) and adaptive (such as T cells) arms of the immune system; on the contrary, cold tumors may be devoid of such an infiltrate [20].

Our results confirmed previous evidence showing that the microenvironment and immune interactions could be fundamental for a proper H-NENs prognostic and therapeutic overview. Robust T-cell infiltration was frequent in GEP-NENs and associated with improved recurrence-free and disease specific survival [12,21]. Hot immune microenvironment with abundant TILS was observed in pancreatic NECs compared to NETs although, in the latter, high intraepithelial PD-1 T cells and PD-L1 Type-II macrophages were observed according to the grade [22]. Moreover, PD-L1 was detected in 10% of H-NENs patients without correlation to progression-free survival or overall survival [23]. Present studies showed that cluster 1 is defined by a high expression of lymphoid T-cells, myeloid infiltrates, HLA antigens and expression of inhibitory receptors and ligands (both PD-1 and PD-L1) in the stroma. T-cell CD3^S^ and CD8^S^, myeloid Arginase^S^ and PD-1^S^ alone as well as clusters, were associated with survival. Cluster 1 included more NET G3 and NEC <55% than cluster 2. Interestingly, survival differences between the two clusters were also observed even by removing the NET G3 (log-rank *p* = 0.016, Supplementary Figure S3). We did not observe a prognostic role for PD-L1. Collectively, our data confirm that innate and adaptive immune cell infiltration has a fundamental role in H-NENs patients and that our cluster 1 could identify the so-called “hot lesions”: H-NENs characterized by a prevalent immune infiltrate cells. Therefore, patients in cluster 1 could potentially be responsive to immunotherapy treatment strategies while, on the contrary, patients in cluster 2, by displaying cold tumors, may less suitable for immunotherapy.

Immune–inflammatory cells represent one of the emerging hallmarks of cancer, due to their role in tumor development [24]. Among the cells of the immune system, the myeloid compartment has been at the center of attention for its role in cancer development; myeloid-derived suppressor cells (MDSCs) and M2 macrophages represent key players in the dynamic process of tumorigenesis [9]. The human MDSCs are characterized by the CD11b^+^CD33^+^HLA-DR^-^ phenotype identifying cell populations with T-cell suppressive activity [25,26]. MDSCs have been associated with worse prognosis in various solid tumors, including gastroenteropancreatic neuroendocrine neoplasms, where MDSCs have been correlated with an advanced cancer stage and the presence of metastasis [27,28,29]. Controversially, recent evidence has shown that increased CD33^+^ myeloid cells were associated with better prognosis in triple negative breast cancer [30]. Moreover, work carried out by Kervarrec et al. on in Merkel Cell Carcinomas (MCC) has showed that CD33^+^ and CD163^+^ infiltrates were closely associated with CD8^+^ T-cell infiltrates and that MCC CD33^+^ CD8^+^ were correlated with improved outcome [31]. The authors showed that infiltrating CD33 cells expressed HLA-DR and, thus, they were not likely MDSC. Similarly, in our cohort myeloid markers showed a positive correlation with lymphoid T-cell markers CD3^S^ and CD8^S^. Cluster 1 H-NENs showed the highest myeloid, HLA and lymphoid T-cell markers expression, suggesting that their role could be related to a higher cancer specific survival than patients with reduced expression. Co-expression of T cell and myeloid markers in cluster 1 tumors is in agreement with the heterogeneous immune contexture described to occur in three tumor immune subtypes defined as “would-healing”, “IFN-g dominant” and “inflammatory”(across several histologies) by Thorsson et al. [20] These evidences indicate that a “hot tumor” (a lesion where spontaneous development of immunity has occurred) is not necessarily characterized only/mainly by infiltration of potentially “anti-tumor” T cells and such tumors are also characterized by the strong presence of myeloid cells. Interestingly, clusters 1 also showed high levels of HLA-DR^S^, compared to cluster 2 (*p* = 0.02, Figure 3, Supplementary Table S2). Consequently, in high-grade neuroendocrine neoplasms, the expression of myeloid markers, associated with high levels of HLA-DR^S^, HLA-I^S^, PD-1^S^, PD-L1^S^ and T-cells markers, most likely, could not have immunosuppressive effects as they identified a sub-group of patients with better survival.

We are aware that the present study could be limited by the pure IHC scoring system that, even if validated by expert pathologists (in emolymphopathology and neuroendocrine tumors), could be not able to fully describe the biological features of the interaction between microenvironment and NENs. The current clinical scenario could benefit from this excellent double tiers method with the appropriate further validations, but IHC as a gross screening tool for inquiring the microenvironment–tumor relationship could be proposed [15,32]. Furthermore, in order to deeply understand the aforesaid interaction, the precise antibody co-localization could have been better if evaluated first by digital pathology tools and then by molecular analysis. Moreover, the present results highlight that the H-NENs heterogeneity is driven also by the microenvironment role, paving the way to further distinguishing H-NENs in two clearly defined prognostic sub classes.

In conclusion, due to the aforementioned H-NENs, heterogeneity and dichotomy, microenvironment-related immune and inflammatory markers can provide helpful information to predict NEN clinical outcomes that could help to select the proper therapeutic approach and hopefully pave the way to novel ones.

## Figures and Tables

**Table 1:** Clinicopathological features of the 45 patients included in the study.

Features	All Patients
Total	45 (100)
Gender	
M	28 (62.2)
F	17 (37.8)
Years	
Median (range)	61 (33–78)
Morphology	
NET G3	6 (13.3)
NEC-Ki67 <55	8 (17.8)
NEC-Ki67 >55	31 (68.9)
Stage	
I-II	3 (6.7)
III	14 (31.1)
IV	28 (62.2)
Site	
Colon	8 (17.8)
Ileum	7 (15.6)
Pancreas	15 (33.3)
Rectum	10 (22.2)
Stomach	5 (11.1)

Abbreviations: NET, neuroendocrine tumor; NEC, neuroendocrine carcinoma; Ki-67, Ki-67 proliferative index.

## Supplementary Materials

The following are available online at https://www.mdpi.com/article/10.3390/jcm10081741/s1, Table S1: Antibody sources and dilutions. Table S2: Clinicopathological, stromal and tumor markers in the two clusters. Table S3: Univariate and multivariable analysis (based on selected variables) of overall survival of patients with High-grade Neuroendocrine Neoplasms. Figure S1: IHC analyses for Arginase, CD163, CD33 and CD66 classified in low (score up to 3), moderate (score 4-8) and high (score 9-12) expression in representative neuroendocrine carcinoma cases. Figure S2: Cancer specific survival of patients with High-grade neuroendocrine neoplasms. Figure S3: Cancer specific survival of patients with poorly differentiated neuroendocrine neoplasms (NEC) according cluster
