# Supplementary material for: Myeloid and T-Cell Microenvironment Immune Features Identify Two Prognostic Sub-Groups in High-Grade Gastroenteropancreatic Neuroendocrine Neoplasms
Source: J Clin Med. 2021 Apr 17;10(8):1741. doi: 10.3390/jcm10081741 (PMC8072982; doi:10.3390/jcm10081741)
Supplement: Supplementary file 1 [file jcm-10-01741-s001.zip › Supplementary Table 1.docx]

| **Supplementary Table 1.** Antibody sources and dilutions. | | | | |
| --- | --- | --- | --- | --- |
| **Antigens** | **Pretratretment** | **Dilution** | **Clone** | **Source** |
| Arginase | pH9 | 1/200 | Polyclonal | Genetex |
| CD33 | pH9 | 1/100 | PWS44 | Leica Biosystems |
| CD163 | pH6 | 1/100 | 10D6 | Leica Biosystems |
| CD66 | pH6 | Ready to use | BY114 | Biogenex |
