# Supplementary material for: Myeloid and T-Cell Microenvironment Immune Features Identify Two Prognostic Sub-Groups in High-Grade Gastroenteropancreatic Neuroendocrine Neoplasms
Source: J Clin Med. 2021 Apr 17;10(8):1741. doi: 10.3390/jcm10081741 (PMC8072982; doi:10.3390/jcm10081741)
Supplement: Supplementary file 1 [file jcm-10-01741-s001.zip › Supplementary Table 2.docx]

| **Supplementary Table 2**: Clinicopathological, stromal and Tumor markers in the two clusters | | | | | | | |
| --- | --- | --- | --- | --- | --- | --- | --- |
|  | | | | | | | |
|  |  |  |  | **All patients** | **Cluster 1** | **Cluster 2** | **P-value*** |
|  |  |  |  |  |  |  |  |
|  |  |  | **Total** | 45 (100) | 22 (100) | 23 (100) |  |
| **Clinicopathological parameters** | |  | **Sex** |  |  |  |  |
|  |  |  | Male | 28 (62.2) | 14 (63.6) | 14 (60.9) |  |
|  |  |  | Female | 17 (37.8) | 8 (36.4) | 9 (39.1) | 1.00 |
|  |  |  | **Years** |  |  |  |  |
|  |  |  | Mean [range] | 61.1 [33-78] | 63.1 [47-78] | 59.1 [33-76] | 0.30 |
|  |  |  | **Site** |  |  |  |  |
|  |  |  | Colon | 8 (17.8) | 4 (18.2) | 4 (17.4) |  |
|  |  |  | Ileum | 7 (15.6) | 5 (22.7) | 2 (8.7) |  |
|  |  |  | Pancreas | 15 (33.3) | 7 (31.8) | 8 (34.8) |  |
|  |  |  | Rectum | 10 (22.2) | 3 (13.6) | 7 (30.4) |  |
|  |  |  | Stomach | 5 (11.1) | 3 (13.6) | 2 (8.7) | 0.50 |
|  |  |  | **Stage** |  |  |  |  |
|  |  |  | I-II | 3 (6.7) | 2 (9.1) | 1 (4.4) |  |
|  |  |  | III | 14 (31.1) | 7 (31.8) | 7 (40.4) |  |
|  |  |  | IV | 28 (62.2) | 13 (59.1) | 15 (65.2) | 0.90 |
|  |  |  | **Morphology** |  |  |  |  |
|  |  |  | NET G3 | 6 (13.3) | 5 (22.7) | 1 (4.4) |  |
|  |  |  | NEC<55 | 8 (17.8) | 5 (22.7) | 3 (13.0) |  |
|  |  |  | NEC>55 | 31 (68.9) | 12 (54.6) | 19 (82.6) | 0.09 |
|  |  |  | **Ki-67** |  |  |  |  |
|  |  |  | <55 | 14 (31.1) | 4 (17.4) | 10 (45.5) |  |
|  |  |  | >55 | 31 (68.9) | 19 (82.6) | 12 (54.5) | 0.06 |
| **Stromal Markers** | Mieloid |  | **Arginase S** |  |  |  |  |
|  |  |  | Mean [range] | 4.89 [0-12] | 5.95 [3-12] | 3.87 [0-9] | **0.002** |
|  |  |  | **CD33 S** |  |  |  |  |
|  |  |  | Mean[range] | 2.71 [0-9] | 3.90 [1-9] | 1.57 [0-4] | **<0.0001** |
|  |  |  | **CD163 S** |  |  |  |  |
|  |  |  | Mean [range] | 4.74 [2-12] | 5.64 [3-12] | 3.87 [2-6] | **0.008** |
|  |  |  | **CD66 S** |  |  |  |  |
|  |  |  | Mean [range] | 1.36 [0-6] | 1.77 [0-6] | 0.96 [0-3] | 0.11 |
|  | T-cell |  | **CD3 S** |  |  |  |  |
|  |  |  | Mean [range] | 3.82 [0-12] | 6.95 [1-12] | 0.83 [0-4] | **<0.0001** |
|  |  |  | **CD4 S** |  |  |  |  |
|  |  |  | Mean [range] | 0.87 [0-9] | 1.73 [0.9] | 0 [0-1] | **0.001** |
|  |  |  | **CD8 S** |  |  |  |  |
|  |  |  | Mean [range] | 2.02 [0-6] | 3.73 [1-6] | 0.39 [0-2] | **<0.0001** |
|  | Immune Checkpoint |  | **PD-L1 S** |  |  |  |  |
|  |  |  | Mean [range] | 1.31 [0-6] | 1.95 [0-6] | 0.7 [0-6] | **0.01** |
|  |  |  | **PD-1 S** |  |  |  |  |
|  |  |  | Mean [range] | 2.93 [0-9] | 4.82 [0-9] | 1.13 [0-6] | **0.0002** |
|  | HLA |  | **HLA-1 S** |  |  |  |  |
|  |  |  | Mean [range] | 2.84 [0-12] | 4.27 [0-12] | 1.48 [0-9] | **0.01** |
|  |  |  | **HLA-DR S** |  |  |  |  |
|  |  |  | Mean [range] | 7.71 [0-12] | 9.05 [2-12] | 6.43 [0-12] | **0.02** |
|  | Nonimmune markers |  | **NGFR S** |  |  |  |  |
|  |  |  | Mean [range] | 1.91 [0-12] | 2.14 [0-12] | 1.70 [0-12] | 0.81 |
|  |  |  | **COX2 S** |  |  |  |  |
|  |  |  | Mean [range] | 2.31 [0-12] | 3.27 [0-12] | 1.39 [0-9] | 0.16 |
|  |  |  | **S6 S** |  |  |  |  |
|  |  |  | Mean [range] | 2.16 [0-12] | 2.77 [0-12] | 1.57 [0-12] | 0.28 |
|  |  |  | **CD31 S** |  |  |  |  |
|  |  |  | Absent | 23 (51.1) | 10 (45.5) | 13 (56.5) |  |
|  |  |  | Present | 22 (48.9) | 12 (54.5) | 10 (43.5) | 0.56 |
| **Tumor Markers** | |  | **COX2T** |  |  |  |  |
|  |  |  | Mean [range] | 9.64 [0-12] | 9.27 [0-12] | 10 [0-12] | 0.59 |
|  |  |  | **S6 T** |  |  |  |  |
|  |  |  | Mean [range] | 2.73 [0-12] | 2.77 [0-12] | 2.70 [0-12] | 0.29 |
|  |  |  | **Beta-Cat T** |  |  |  |  |
|  |  |  | Mean [range] | 9.49 [0-12] | 9.00 [0-12] | 9.96 [0-12] | 0.54 |
|  |  |  | **HLA-1 T** |  |  |  |  |
|  |  |  | Mean [range] | 5.51 [0-12] | 6.04 [0-12] | 5.00 [0-12] | 0.51 |
|  |  |  | **PD-L1 T** |  |  |  |  |
|  |  |  | Mean [range] | 0.4 [0-9] | 0.82 [0-9] | 0.0 [0-0] | 0.15 |
|  |  |  | **CD31 T** |  |  |  |  |
|  |  |  | Absent | 24 (46.7) | 8 (36.4) | 13 (56.5) |  |
|  |  |  | Present | 24 (53.3) | 14 (63.6) | 10 (43.5) | 0.24 |
|  |  |  | **Beta-Cat T** |  |  |  |  |
|  |  |  | Absent | 2 (4.4) | 1 (4.6) | 1 (4.4) |  |
|  |  |  | Cytoplasmic and/or membrane | 25 (55.6) | 14 (63.6) | 11 (47.8) |  |
|  |  |  | Nuclear | 18 (40.0) | 7 (31.8) | 11 (47.8) | 0.67 |
|  |  |  |  |  |  |  |  |
|  | * P-value based on the Fisher Exact test for categorical variables or the Wilcoxon test for continuous variables. | | | | | | |
