# Supplementary material for: Myeloid and T-Cell Microenvironment Immune Features Identify Two Prognostic Sub-Groups in High-Grade Gastroenteropancreatic Neuroendocrine Neoplasms
Source: J Clin Med. 2021 Apr 17;10(8):1741. doi: 10.3390/jcm10081741 (PMC8072982; doi:10.3390/jcm10081741)
Supplement: Supplementary file 1 [file jcm-10-01741-s001.zip › Supplementary Table 3.docx]

| **Supplementary Table 3:** Univariate and multivariable analysis (based on selected variables) of overall survival of patients with High-grade Neuroendocrine Neoplasms | | | | | | |
| --- | --- | --- | --- | --- | --- | --- |
| **Variable** | **Univariate HR (95% CI)** | **P-value** | **Adjusted for Site HR (95% CI)** | **P-value** | **Multivariable HR (95% CI)** | **P-value** |
| **Gender** (Male vs Female) | 0.83 (0.44-1.57) | 0.57 |  |  |  |  |
| **Years** (Increase of 10-years) | 1.27 (0.91-1.78) | 0.17 |  |  |  |  |
| **Site** |  |  |  |  |  |  |
| Colon-rectum | 1.00 |  |  |  | 1.00 |  |
| Stomach | 0.64 (0.23-1.77) | 0.39 |  |  | 0.94 (0.33-2.67) | 0.91 |
| Ileum | 0.26 (0.10-0.69) | 0.007 |  |  | 0.37 (0.13-1.04) | 0.06 |
| Pancreas | 0.28 (0.13-0.62) | 0.002 |  |  | 0.51 (0.21-1.23) | 0.13 |
| **Stage** (IV vs I-II-III) | 1.18 (0.59-2.36) | 0.63 |  |  |  |  |
| **Ki-67** (>55 vs <55) | 13.30 (4.40-40.23) | <0.0001 | 10.7 (3.34-34.26) | <0.0001 | 8.60 (2.61-28.33) | <0.0001 |
| **Cluster** (1 vs 2) | 0.36 (0.18-0.73) | 0.005 | 0.30 (0.14-0.63) | 0.001 | 0.43 (0.20-0.93) | 0.03 |
| **Arginase S** (4-12 vs 0-3) | 0.34 (0.16-0.73) | 0.006 | 0.40 (0.18-0.91) | 0.03 |  |  |
| **CD33 S** (3-12 vs 0-2) | 0.64 (0.33-1.24) | 0.19 |  |  |  |  |
| **CD163 S** (4-12 vs 0-3) | 1.45 (0.66-3.20) | 0.36 |  |  |  |  |
| **CD66 S** (1--12 vs 0) | 0.76 (0.39-1.45) | 0.40 |  |  |  |  |
| **CD3 S** (3-12 vs 1-2) | 0.33 (0.16-0.65) | 0.002 | 0.31 (0.15-0.64) | 0.001 |  |  |
| **CD4 S** (1-12 vs 0) | 0.65 (0.31-1.40) | 0.27 |  |  |  |  |
| **CD8 S** (2-12 vs 0-1) | 0.49 (0.25-0.96) | 0.04 | 0.45 (0.22-0.92) | 0.03 |  |  |
| **PD-L1 S** (1-12 vs 0) | 1.00 (0.53-1.90) | 0.99 |  |  |  |  |
| **PD-1 S** (2-12 vs 0-1) | 0.52 (0.28-0.98) | 0.04 | 0.33 (0.15-0.71) | 0.005 |  |  |
| **HLA-1 S** (1-12 vs 0) | 0.78 (0.41-1.48) | 0.45 |  |  |  |  |
| **HLA-DR S** (12 vs 0-9) | 0.74 (0.35-1.55) | 0.43 |  |  |  |  |
| **NGFR S** (1-12 vs 0) | 0.99 (0.49-1.99) | 0.98 |  |  |  |  |
| **COX2 S** (1-12 vs 0) | 0.87 (0.45-1.70) | 0.68 |  |  |  |  |
| **S6 S (1-12 vs 0)** | 0.91 (0.45-1.82) | 0.79 |  |  |  |  |
| **CD31 S** (Present vs Absent) | 1.86 (0.97-3.58) | 0.06 |  |  |  |  |
| **COX2T** (12 vs 0-9) | 0.71 (0.38-1.35) | 0.30 |  |  |  |  |
| **S6 T** (1-12 vs 0) | 0.49 (0.26-0.94) | 0.03 |  |  |  |  |
| **Beta-Cat T** (12 vs 0-9) | 1.27 (0.66-2.44) | 0.48 |  |  |  |  |
| **HLA-1 T** (6-12 vs 0-4) | 0.93 (0.50-1.80) | 0.83 |  |  |  |  |
| **PD-L1 T (**1-12 vs 0**)** | 2.47 (0.57-10.81) | 0.23 |  |  |  |  |
| **CD31 T** (Present vs Absent) | 1.82 (0.94-3.52) | 0.08 |  |  |  |  |
| **Beta-Cat T** |  |  |  |  |  |  |
| Absent | 1.00 |  |  |  |  |  |
| Cytoplasmic and/or membrane | 0.37 (0.08-1.64) | 0.19 |  |  |  |  |
| Nuclear | 0.68 (0.16-2.99) | 0.61 |  |  |  |  |
