# Supplementary figures and images for: Myeloid and T-Cell Microenvironment Immune Features Identify Two Prognostic Sub-Groups in High-Grade Gastroenteropancreatic Neuroendocrine Neoplasms
Source: J Clin Med. 2021 Apr 17;10(8):1741. doi: 10.3390/jcm10081741 (PMC8072982; doi:10.3390/jcm10081741)

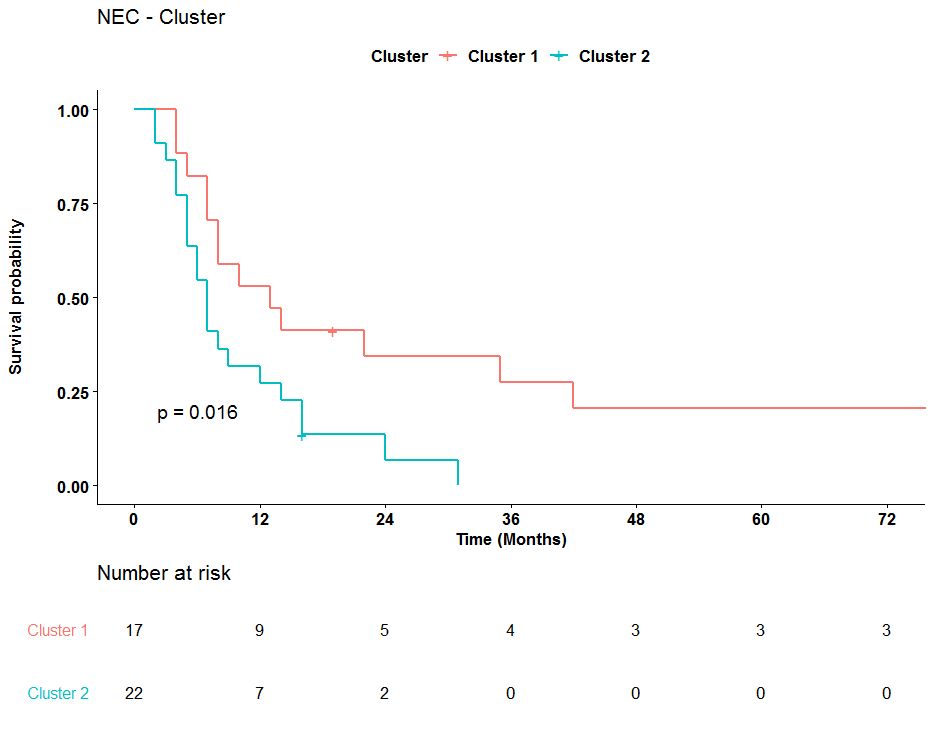

Supplement: Supplementary file 1 [file jcm-10-01741-s001.zip › Supplementary Figure 3.png]

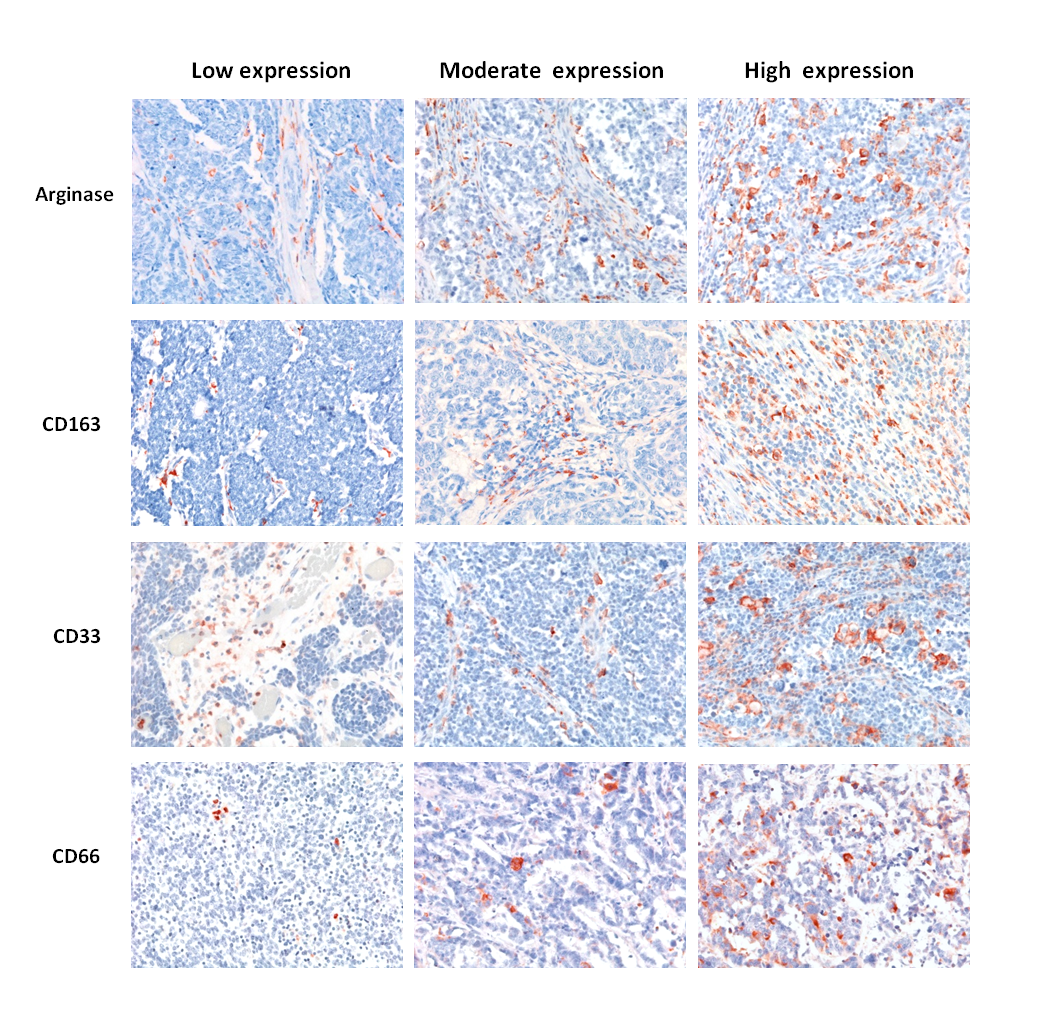

Supplement: Supplementary file 1 [file jcm-10-01741-s001.zip › Supplementary Figure 1.png]

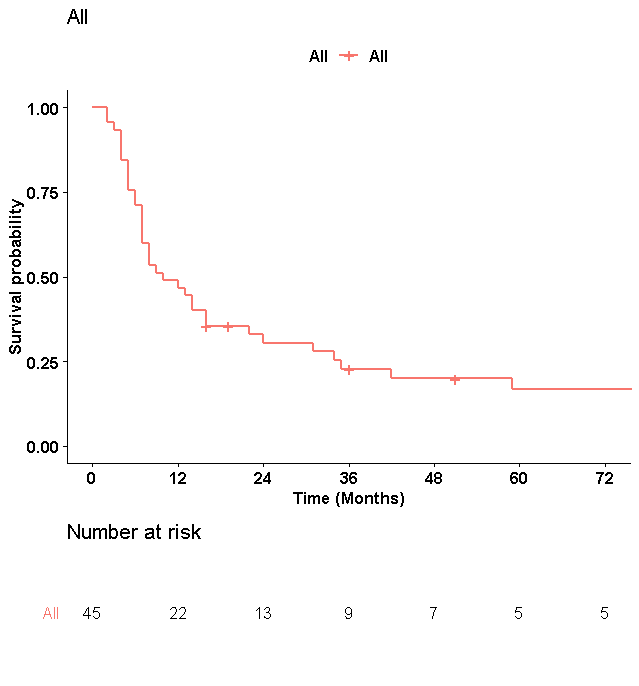

Supplement: Supplementary file 1 [file jcm-10-01741-s001.zip › Supplementary Figure 2.png]
